# Lower limb muscle activation and biomechanics during single-leg hopping in different directions

**DOI:** 10.3389/fspor.2025.1733669

**Published:** 2026-01-09

**Authors:** Yu Gu, Wanyan Su, Nawfal Malik, Thanh Nguyen, Anne Jordan, Trent Herda, Yu Song

**Affiliations:** Department of Health, Sport & Exercise Sciences, University of Kansas, Lawrence, KS, United States

**Keywords:** anterior cruciate ligament, ACL injury, knee, kinematic, kinetic, rehabilitation

## Abstract

**Background:**

Single-leg forward and vertical hopping are commonly employed to evaluate knee neuromuscular function following anterior cruciate ligament reconstruction. However, similar hopping performance between limbs does not ensure full knee recovery. Single-leg backward hopping has been suggested to impose greater knee kinetics, but its effects on lower limb muscle activation and kinematics remain unclear.

**Purpose:**

To quantify the effect of hopping directions on lower limb muscle activation and biomechanics during jumping, focusing on the knee joint.

**Methods:**

Forty-eight injury-free participants performed single-leg forward/vertical/backward hopping with motion, force, and surface electromyography data collected. Peak and mean muscle activation of quadriceps, hamstrings, and triceps surae, peak trunk/hip/knee/ankle angles, and hip/knee/ankle moments in the sagittal plane during the jumping phase were calculated. Hopping performance was also recorded. One-by-three repeated-measures ANOVAs were conducted to quantify the effects of hopping directions.

**Results:**

Forward hopping demonstrated greater hopping performance, trunk/ankle angles, hip/ankle moments, and hamstring activations, while smaller hip/knee angles, knee moments, and quadriceps activations compared to hopping in other directions. Vertical hopping showed the greatest knee angle compared to forward and backward hopping. Backward hopping exhibited the smallest trunk/ankle angles, hip/ankle moments, and muscle activations of biceps femoris/gastrocnemius medialis/soleus with the greatest knee moment among hopping directions.

**Conclusion:**

Forward hopping may serve as a general performance exercise but might underrepresent knee-specific measurements. Vertical hopping may be more appropriate for monitoring quadriceps function and knee control. Backward hopping imposes the greatest knee mechanical demands with limited hip and ankle involvement, likely making it a promising metric for identifying deficits in dynamic knee control.

## 1 Introduction

Anterior cruciate ligament (ACL) injury is associated with prolonged absence from playing (1), long-term deficits in neuromuscular function (2), and an elevated risk of reinjury (3). Despite the frequent employment of criterion-based testing batteries to evaluate knee neuromuscular function and support return-to-play decisions after ACL reconstruction (ACLR) (4), nearly one-fourth of patients sustain a secondary ACL injury (1). This high ACL reinjury risk highlights the need for more effective assessments to monitor rehabilitation progress and contribute to safely returning to play.

Bilateral asymmetries in knee neuromuscular function are common post-ACLR, characterized by quadriceps weakness and decreased knee extension moments in the ACL-affected limb compared to the unaffected limb (5–7). Functional performance tasks like single-leg forward hopping for distance and vertical hopping for height are widely employed to evaluate knee neuromuscular function due to their practicality (4, 8). A common benchmark used for returning to play is that the ACL-affected limb can achieve at least 90% of the hopping performance of the unaffected limb (5, 9). However, previous studies show that achieving symmetrical hopping performance does not necessarily indicate fully restored knee function. For instance, despite demonstrating 97% symmetry of forward hopping performance, the ACL-affected limb produced only 69% of the knee kinetics of the unaffected limb and relied more on hip and ankle engagement to compensate during the jumping phase (10). Additionally, while greater asymmetry has been reported in vertical hopping height than in forward hopping distance (11), limited agreement was shown between hopping performance and quadriceps strength in both tasks (5, 12, 13). For example, forward and vertical hopping performance explain approximately 40% and 30% of the variance in quadriceps strength, respectively (5, 12, 13). Therefore, commonly used hopping tasks in clinics provide a limited representation of knee function following ACLR, highlighting the need for additional assessments that evaluate the knee joint more directly.

Given the limitations of traditional forward and vertical hopping tasks, recent work placed attention toward hopping in another direction, backward (14). Single-leg backward hopping for distance has been proposed as a more knee-demanding task compared to forward and vertical hopping, demonstrating greater knee kinetics and smaller power and moments at hips and ankles during jumping (14, 15). These findings suggest backward hopping might be a more appropriate or at least an additional metric to assess knee function during rehabilitation after ACLR. However, while previous studies have reported lower-limb kinetics such as work, power, and joint moments during the jumping phase, the corresponding kinematic patterns remain unclear. Since regaining full knee range of motion is a critical rehabilitation milestone after ACLR (16), evaluating both lower limb joint angles and kinetics during jumping may provide a more comprehensive understanding to guide rehabilitation progression.

Muscles crossing the knee are also critical in altering lower limb kinematics and kinetics (17), which is associated with knee function (11). The primary ACL loading mechanism occurs in the sagittal plane, consisting of the anterior tibial shear force applied to a close-to-full extended knee (18, 19). Major muscle groups impacting this mechanism include quadriceps, hamstrings, and triceps surae. While most studies quantifying musculature in patients following ACLR have focused on quadriceps and hamstrings (20, 21), the triceps surae also contributed to knee joint loading during walking and jump-landings (10, 17). ACLR patients demonstrated greater muscle force of biceps femoris (BF) and gastrocnemius lateralis (GL) while smaller muscle force of soleus compared to healthy individuals during the jumping phase of both forward and vertical hopping (10, 11). Although muscle force has been estimated in these traditional hopping tasks, limited investigations have quantified lower limb muscle activation patterns across forward, vertical, and backward hopping to allow a more comparable assessment of knee neuromuscular function.

To date, limited research has quantified differences in both muscle activation and biomechanics between forward and vertical hopping (22, 23). In healthy individuals, vertical hopping showed significantly greater peak muscle activation of rectus femoris (RF) but smaller activation of BF compared to forward hopping (22). Similarly, greater RF and smaller BF contributions were observed during the jumping phase of vertical hopping in relation to forward hopping (23). Meanwhile, significantly greater ankle flexion angles, smaller peak knee flexion angles, and smaller peak hip moments in the sagittal plane were found in forward hopping compared to vertical hopping (22, 23). These findings suggest that hopping directions affected knee neuromuscular function through lower limb muscle activations, kinematics, and kinetics. Yet, muscle activation and biomechanics of single-leg backward hopping remain unknown. Characterizing lower limb muscle activation, kinematics, and kinetics across various hopping directions may help identify task-specific demands and enhance the utility of hopping tasks in assessing knee neuromuscular function.

This study aimed to determine the effects of hopping directions (forward/vertical/backward) on lower limb (hip/knee/ankle) muscle activations and biomechanics, with a focus on the knee joint. As the first step to quantify lower limb muscle activation and biomechanics during single-leg hopping tasks, injury-free participants were recruited. It was hypothesized that single-leg forward hopping would show the smallest knee flexion angles, knee moments, and quadriceps activation but the greatest hamstring activation compared to both vertical and backward hopping. It was also hypothesized that single-leg backward hopping would demonstrate the greatest knee moment, the lowest hip and ankle moments, and the greatest quadriceps activation among the three tasks.

## 2 Materials and methods

### 2.1 Participants

The smallest estimated effect size for peak RF and BF activation between forward and vertical hopping was 0.62 (22). The smallest reported effect size of knee moment among single-leg hopping directions was 0.80 (14). Given the smallest effect size of 0.62, a sample size of 23 was needed to achieve 80% power at a type I error of 0.05. Forty-eight healthy individuals (30 females and 18 males, age: 20.9 ± 0.7 years old, height: 1.7 ± 0.1 m, and mass: 68.1 ± 12.0 kg) were recruited in the current study. Inclusion criteria were 1) participation in physical exercise at least twice per week, totaling a minimum of 2–3 h per week at the time of testing; 2) no trunk/lower limb surgery history; 3) no injuries restricting participation in physical activities for more than two weeks in the past six months; 4) no condition preventing maximal-effort physical activities; and 5) prior experience in sports involving jump-landing activities, such as basketball, soccer, volleyball, etc. (14). This study was approved by the University of Kansas Institutional Review Board. Participants signed a consent form prior to data collection.

### 2.2 Protocol

**Figure 1:**
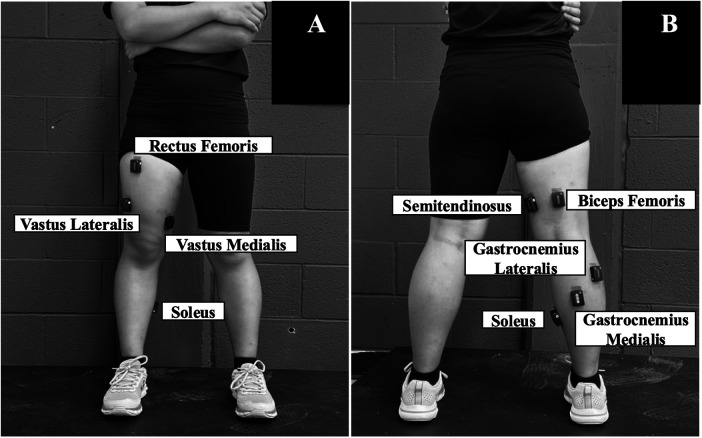
Anterior **(A)** and posterior **(B)** close-up views of electromyography electrode placement on the quadriceps, hamstrings, and triceps surae.

Participants completed a warm-up protocol (14). Eight electromyography (EMG) electrodes (Trigno Avanti system, Delsys Inc., Boston, MA, USA; 2,040 Hz) were placed on the quadriceps [vastus lateralis (VL), RF, vastus medialis (VM)], hamstring (BF and semitendinosus), and triceps surae [GL, gastrocnemius medialis (GM), soleus] of the designated jumping leg, following SENIAM guidelines (Figure 1) (24). The jumping leg was predetermined using a counterbalanced order among participants (15). The skin was shaved and cleaned using alcohol pads to reduce impedance prior to placement. All sensors were taped to the skin to reduce noise.

Then, maximum voluntary isometric contraction (MVIC) was recorded for each muscle group. Quadriceps were tested with participants seated with an external knee flexion of 60° (11); hamstrings were tested prone on a mat with knee flexed at the same angle; triceps surae were tested in single-leg stance with a fully extended knee and maximal plantar flexion (25). Participants pushed as hard as possible for 5 s with one MVIC trial per muscle group in a randomized order. A minimum of 30 s of rest in between MVIC tests was controlled to avoid fatigue.

Next, sixteen retroreflective markers were placed on the participants' super sternal, bilateral acromioclavicular joints and greater trochanters, anterior thigh, lateral and medial femoral condyles, tibial tuberosity, inferior shank, lateral and medial malleolus, tip of halluces, first and fifth metatarsal heads, and heel of the jumping leg. After the static trial, participants performed single-leg forward, vertical, and backward hopping with the order counterbalanced across participants. For the forward and backward hopping, participants jumped from a force plate (Bertec FP6090-15-TM-2000, Columbus, OH, USA, 1,200 Hz) either forward or backward for maximal distance and landed on the same leg. For the vertical hopping, participants jumped maximally for height and landed on the same leg. For all tasks, the non-jumping leg was held at ∼90° of hip/knee flexion during jumping, balance was maintained for at least 3 s after landing, and hands were on the hips. A trial was considered successful if it met all requirements. Five familiarization practices and five successful trials were performed for each task. Verbal encouragement was provided throughout data collection to promote maximal effort. At least 15 s of rest was given between trials to minimize potential fatigue. Three-dimensional marker trajectories were captured using eight opto-reflective cameras (Vicon Vero v2.2, Oxford, UK, 120 Hz), which synchronized with EMG and force plate systems.

### 2.3 Data reduction

EMG signals were filtered using a fourth-order Butterworth bandpass filter of 20–450 Hz (26). Filtered signals were then rectified and lowpass filtered using a fourth-order Butterworth filter at a cut-off of 10 Hz to obtain linear envelopes. The peak value of the processed signal in the MVIC testing was extracted for each muscle. The muscle activation level during the hopping tasks was then normalized to the corresponding MVIC value and expressed as a percentage (25). Mean and peak activation were calculated for each muscle during the jumping phase, defined from the lowest hip position to takeoff (impact force <20 N).

Marker and force data were filtered using a fourth-order Butterworth filter with a lowpass cut-off of 15 Hz for the inverse dynamic approach (27). The hip, knee, and ankle centers were defined following previous studies (28). A trunk segment reference was defined using bilateral acromioclavicular joints and the midpoint of bilateral greater trochanters. A thigh segment reference frame was defined using the hip joint center, knee joint center, and lateral femoral condyle. A shank segment reference frame was defined using the knee joint center, ankle joint center, and lateral malleolus. A foot segment reference frame was determined using the heel, the tip of the hallux, and the fifth metatarsal head. Hip, knee, and ankle flexion angles were calculated as Cardan angles between adjacent segment references (29), while the trunk flexion angle was calculated relative to the global reference (26). The internal resultant joint moments were calculated in the sagittal plane using a custom bottom-up inverse dynamics approach (30) and normalized to the product of body weight and body height. Forward and backward hopping distances were calculated as the displacement of the tip of the hallux and heel markers in the respective hopping direction (14). Vertical hopping height was determined using the midpoint of the bilateral greater trochanters, defined as the difference between peak jump height and standing height (14).

The independent variables were three hopping directions. The dependent variables included lower limb muscle activations and biomechanical parameters. The muscle activation included peak and mean activation of eight individual muscles during jumping. The biomechanical parameters involved hopping performance, jumping duration, peak trunk, hip, knee, and ankle flexion angle during jumping, and peak hip, knee, and ankle moments in the sagittal plane during jumping. The jumping phase was selected because it places a significant mechanical demand on the lower limb and provides an indication of how the knee and surrounding muscles engage during functional and weight-bearing movement (11, 31). The average among five official trials was reported and used for statistical analysis. All data processing was performed in MATLAB 2024a (MathWorks Inc., Natick, MA, USA).

### 2.4 Statistical analysis

One-by-three repeated-measures analyses of variance (ANOVA) were conducted to quantify the effects of hopping directions on lower limb muscle activations and biomechanics. The Type I error rate was set at 0.05 for statistical significance. Paired t-tests were performed for *post hoc* comparisons when a significant main effect was observed. The Benjamini-Hochberg procedure was applied to all pairwise comparisons to control the study-wise false discovery rate at 0.05 (32). Effect sizes for paired comparisons were calculated using Cohen's dz, with Cohen's dz ≤ 0.5 indicating “small,” 0.5 < Cohen's dz < 0.8 indicating “medium,” and Cohen's dz ≥ 0.8 indicating “large” (33). Statistical analyses were performed using SPSS 29.0 (IBM Corporation, Armonk, NY, USA).

## 3 Results

Significant main effects among hopping directions were observed in all dependent variables except for peak muscle activation of VM and GL. Overall, 71 paired *t*-tests were performed with the largest *p*-value of 0.019 after the Benjamini-Hochberg adjustment. Descriptive statistics of muscle activations are reported in Supplementary Appendix S1, while effect sizes and *p*-values for paired comparisons are shown in Supplementary Appendices S2, S3.

**Figure 2:**
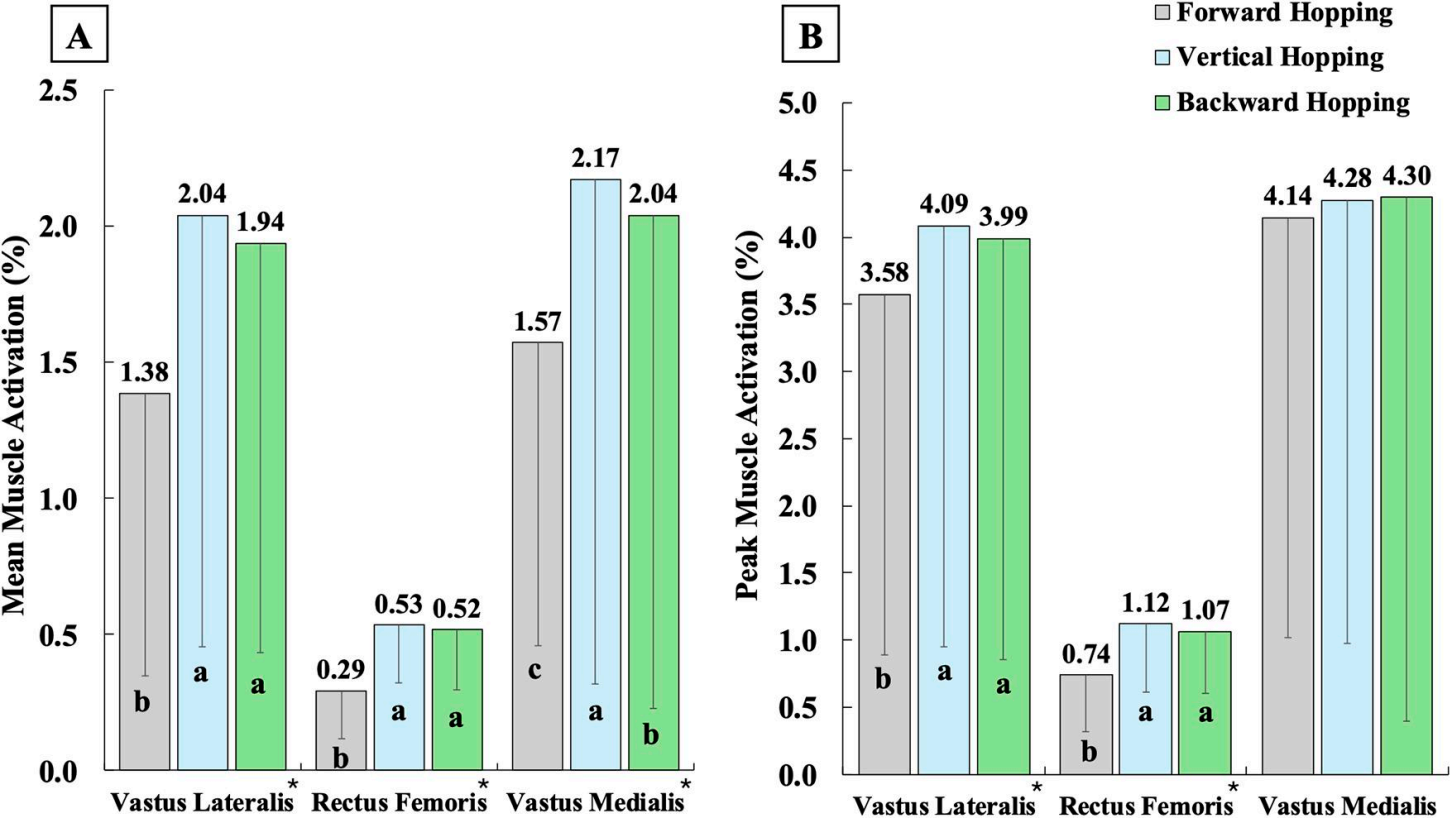
Mean **(A)** and peak **(B)** muscle activations of quadriceps during the jumping phase in single-leg forward (grey), vertical (blue), and backward (green) hopping. * indicates significantly different among the three hopping directions, with a being the greatest, b being the second greatest, and c being the least.

**Figure 3:**
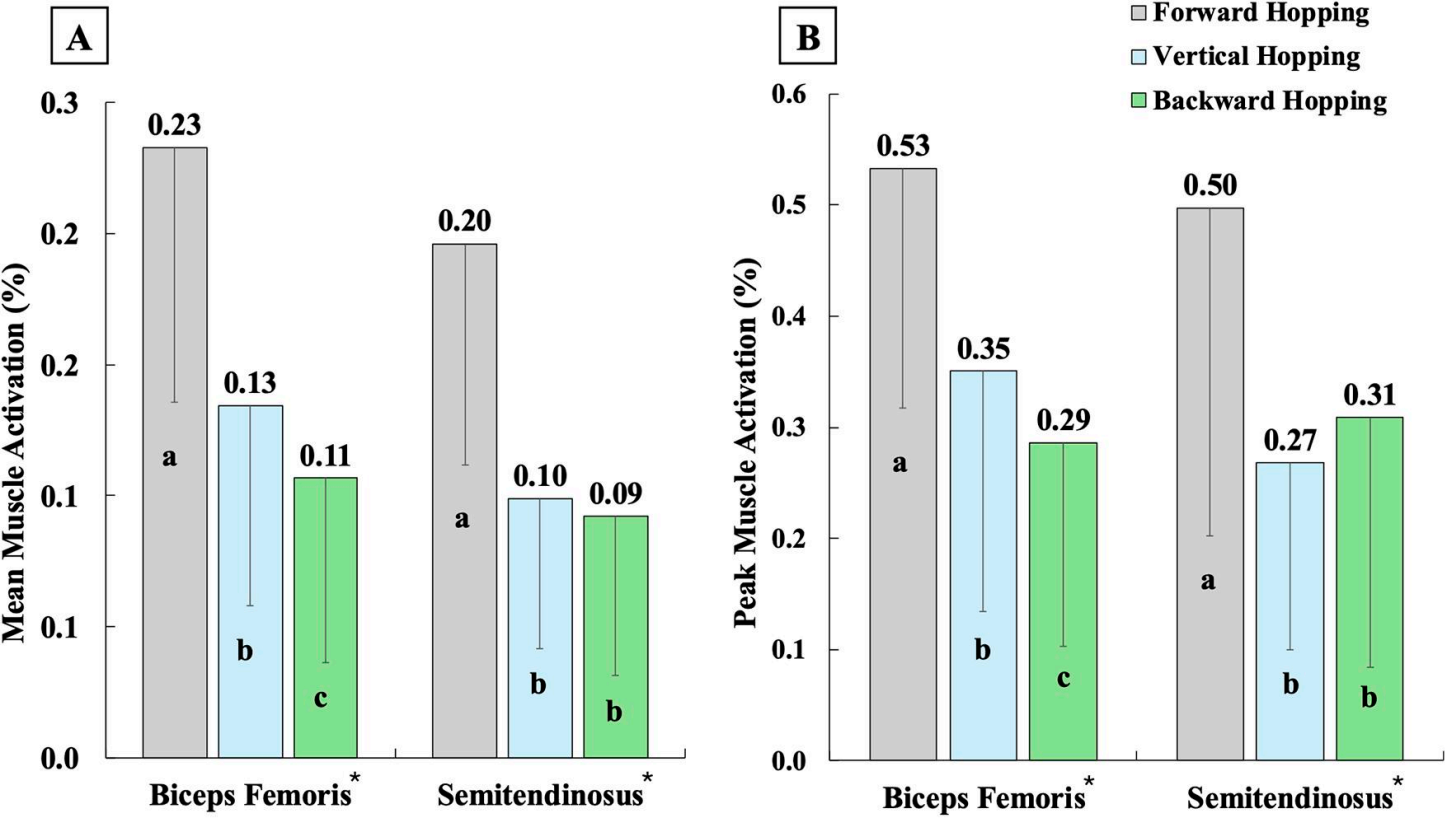
Mean **(A)** and peak **(B)** muscle activations of hamstrings during the jumping phase in single-leg forward (grey), vertical (blue), and backward (green) hopping. * indicates significantly different among the three hopping directions, with a being the greatest, b being the second greatest, and c being the least.

**Figure 4:**
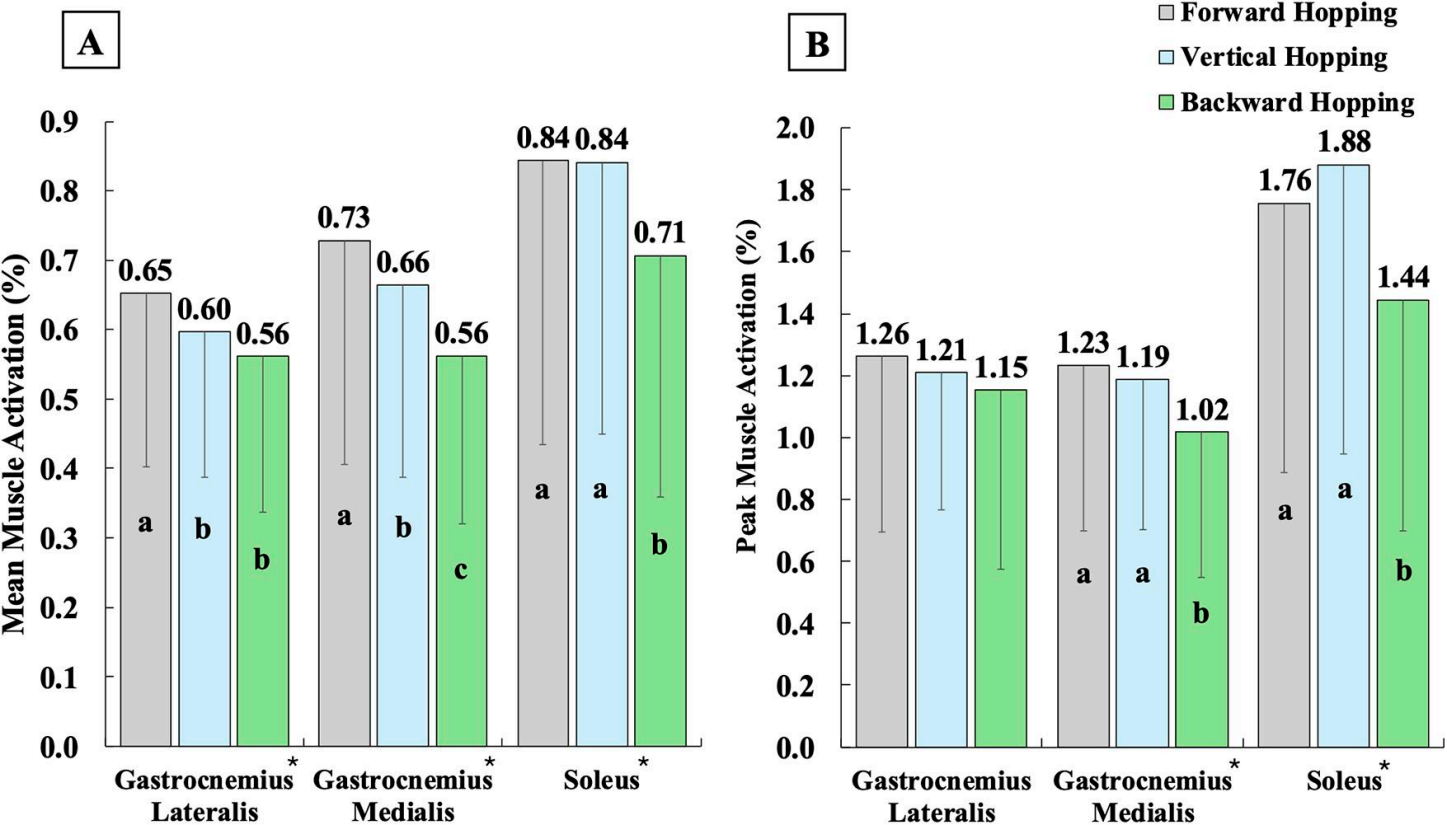
Mean **(A)** and peak **(B)** muscle activations of triceps surae during the jumping phase in single-leg forward (grey), vertical (blue), and backward (green) hopping. * indicates significantly different among the three hopping directions, with a being the greatest, b being the second greatest, and c being the least.

Regarding muscle activations (Figures 2–4), single-leg forward hopping demonstrated the lowest mean activation of VM, the greatest mean activation of BF and GM, and the greatest peak activation of BF, compared to both vertical and backward hopping. Greater mean and peak activations of RF and VL, smaller mean and peak activations of semitendinosus, and smaller mean activation of GL were found in vertical and backward hopping in comparison to forward hopping. Single-leg backward hopping also showed the lowest mean and peak activation of BF, GM, and soleus compared to hopping in other directions.

Regarding biomechanical outcomes (Table 1), peak trunk flexion, peak ankle dorsiflexion, and peak hip and ankle moments showed a consistent pattern, with the greatest values observed in forward hopping, followed by vertical hopping, and the smallest values in backward hopping. Vertical hopping showed the longest jumping duration and greatest knee flexion angle compared to forward and backward hopping. Additionally, Backward hopping exhibited the shortest jumping duration and the greatest knee moment compared to forward and vertical hopping.

**Table 1:** Mean ± standard deviation and *p*-values of main effects observed in repeated-measures ANOVAs in biomechanical parameters.

Biomechanical parameters	Single-leg Forward Hopping	Single-leg Vertical Hopping	Single-leg Backward Hopping	*P*-values of main effect
Hopping performance (m)	1.23 ± 0.29^a^	0.23 ± 0.06^c^	0.81 ± 0.18^b^	**<0** **.** **001**
Jumping duration (s)	0.26 ± 0.11^b^	0.34 ± 0.12^a^	0.23 ± 0.05^c^	**<0**.**001**
Peak trunk flexion angle during jumping (°)	36.6 ± 9.6^a^	28.7 ± 11.9^b^	23.4 ± 9.9^c^	**<0**.**001**
Peak hip flexion angle during jumping (°)	53.7 ± 14.1^b^	62.5 ± 18.8^a^	62.6 ± 15.4^a^	**<0**.**001**
Peak knee flexion angle during jumping (°)	59.6 ± 8.7^c^	67.5 ± 10.4^a^	63.3 ± 8.8^b^	**<0**.**001**
Peak ankle dorsiflexion angle during jumping (°)	32.7 ± 5.2^a^	28.1 ± 4.9^b^	17.3 ± 5.0^c^	**<0**.**001**
Peak hip moment during jumping (BW*BH)	0.200 ± 0.029^a^	0.180 ± 0.036^b^	0.167 ± 0.032^c^	**<0**.**001**
Peak knee moment during jumping (BW*BH)	0.073 ± 0.022^c^	0.097 ± 0.023^b^	0.113 ± 0.022^a^	**<0**.**001**
Peak ankle moment during jumping (BW*BH)	0.151 ± 0.021^a^	0.113 ± 0.017^b^	0.090 ± 0.012^c^	**<0**.**001**

BW, body weight; BH, body height. a, b, and c, significantly different among the three hopping directions.

Statistically significant differences are shown in bold.

a is the greatest.

b is the second greatest.

c is the least.

## 4 Discussion

This study aimed to determine the effects of hopping directions on lower limb muscle activations and biomechanics. The results supported the first hypothesis that single-leg forward hopping demonstrated smaller knee flexion angles, knee extension moments, and quadriceps activation compared to other hopping directions, with medium to large effect sizes. These findings were generally consistent with previous studies (14, 22, 34). Prior work has reported that both single-leg and double-leg forward hopping show greater ankle dorsiflexion angles, greater peak hip and ankle moments, as well as smaller peak knee flexion angle and peak knee extension moment compared to vertical hopping (14, 22, 34). Greater hamstring activation and smaller quadriceps activation have also been observed in forward hopping relative to vertical hopping (22). In the current study, joint flexion angles in single-leg forward (∼40° less trunk/hip flexion, ∼30° less knee flexion, and ∼8° less ankle dorsiflexion) and vertical (∼25° less trunk flexion, ∼30° less hip/knee flexion, and ∼8° less ankle dorsiflexion) hopping were smaller than those reported in double-leg hopping tasks (22) but similar to those reported in previous single-leg hopping studies (34). This pattern likely reflects the increased demands of single-leg tasks, which require greater balance, strength, and control. As a result, participants may have adopted more upright postures with limited joint flexion during jumping (35).

Joint angles during these functional performance tasks represent an individual's ability to control these joints under weight-bearing and closed-kinetic-chain conditions, providing a practical measure of neuromuscular control during jumping. Forward hopping involved less hip flexion than vertical hopping but greater trunk flexion, creating a forward-leaning posture that likely facilitates anterior momentum (22). This trunk positioning likely enhances performance by shifting the center of mass (COM) anteriorly. Biarticular muscles such as RF and BF may coordinate hip and knee movements, while hip and ankle extension generate tangential velocity to propel the body forward. This kinematic strategy agrees with previous studies that forward hopping relies more heavily on hip and ankle contributions, with reduced knee involvement (15, 31). Together, despite forward hopping may challenge balance, strength, and overall performance, its limited knee involvement may restrict its utility as a functional assessment of the knee joint (10, 15, 36).

In addition, the greatest knee flexion angle was reported in single-leg vertical hopping compared to forward and backward hopping. Yet, the differences were small (∼4°–8°), which are unlikely to be clinically detectable. Quadriceps activation was also significantly greater in vertical hopping than in forward hopping, while it did not differ from backward hopping. These results may indicate that vertical hopping engages the knee more extensively than forward hopping, though through a different neuromuscular strategy compared to backward hopping. During vertical hopping, participants appeared to rely heavily on the quadriceps, with simultaneous coactivation of the hamstrings and triceps surae, which is consistent with the greater knee flexion angle observed. In contrast, backward hopping produced similar quadriceps activation but substantially lower hamstring and triceps surae activations, associated with a slightly smaller knee flexion angle (∼4°). Backward hopping also showed the greatest knee moment among the three hopping directions, indicating an excessive net knee engagement. Therefore, both vertical and backward hopping place substantial demand on the knee, but through distinct patterns of muscle engagement.

Previous studies proposed vertical hopping as a more knee-specific alternative compared to forward hopping due to its higher mechanical demands at the knee and greater sensitivity to asymmetries in jump height after ACLR (11, 31). The slightly greater knee flexion angles observed in the current study are consistent with those findings. This pattern may also be related to its biomechanical similarity to common exercises, such as squatting and countermovement jumps, which place high demands on the lower limb in the vertical direction (35, 37). Familiarity with these vertical weight-bearing tasks may allow participants to bend more deeply and prolong the jumping phase. For example, peak knee flexion angles of the ACL-affected limb during squatting have shown moderate associations with peak knee flexion angles during jumping (37), and similar correlations have been reported between squatting and landing knee flexion angles in healthy individuals (35). As such, the combination of greater knee flexion angle and quadriceps activation level supports the potential utility of vertical hopping as a functional task to assess and challenge knee neuromuscular function compared to forward hopping. Vertical hopping may also serve as a partial bridge exercise between controlled strength training and high-speed dynamic movements, facilitating knee control for more explosive and sport-specific activities.

The findings generally supported the second hypothesis that single-leg backward hopping showed the smallest peak trunk/ankle flexion angles, hip/ankle moments, and BF/GM/soleus activation. In contrast, it demonstrated the greatest knee moments among the three hopping directions and greater hip/knee flexion angles than forward hopping, with most differences showing large effect sizes. As the trunk contributes approximately half of the overall body mass, its movement strongly influences the COM (38). The more upright trunk posture observed during backward hopping likely reflects the specific demands of the task. To move in a backward direction, participants must maintain their trunk in a more upright and posterior position, whereas forward and vertical hopping allow or require the trunk to lean forward and flex more to assist in lowering the COM. With the greatest knee moments and the smallest hip/ankle moments found during backward hopping, the results reinforced the previous studies that backward hopping imposes the greatest mechanical demands on the knee (greater knee power/work/moment) compared to forward and vertical hopping (14, 39). Given the small knee flexion differences (∼4°) and similar quadriceps activation between vertical and backward hopping, as well as the reduced hamstring and triceps surae activation during backward hopping, this task may serve as an additional metric for isolating the knee joint and producing the greatest knee moment among the hopping directions.

The current study has implications. The directional specificity of single-leg hopping tasks provides an opportunity to tailor assessments based on the mechanical demands of each task. Forward hopping may serve as a general balance and performance exercise, but it appears to underrepresent knee-specific measurements. In contrast, vertical hopping, with its greater knee flexion and quadriceps engagement, may be more appropriate for monitoring an individual's ability to control the knee under weight-bearing and closed-kinetic-chain conditions. In particular, backward hopping imposes the greatest mechanical demand on the knee with limited hip and ankle involvement, likely making it a promising metric for identifying deficits in dynamic knee control and potentially improving the sensitivity of return-to-play evaluations. Incorporating these task-specific features into training and evaluation protocols may contribute to more effective rehabilitation monitoring.

There were several limitations. Although this study established a baseline understanding of lower limb muscle activation and biomechanical patterns across hopping directions in injury-free populations, the generalizability of the findings was limited to ACLR patients. Investigating the lower limb muscle activation and biomechanics during backward hopping in comparison to traditional forward/vertical hopping in ACLR patients is needed. Second, while this study quantified joint-level kinematics and kinetics, it did not directly estimate muscle force or evaluate the position of the whole-body and/or segmental COM. Understanding the spatial relationship between the COM and the force vector could provide deeper insight into the mechanical strategies underlying each hopping direction. Third, this study did not measure gluteus activation, which plays an important role in hip extension and trunk-pelvis stabilization. Involving the gluteus activations would contribute to the hip joint during dynamic single-leg hopping tasks.

## 5 Conclusions

Single-leg forward hopping primarily relied on hip and ankle contributions, suggesting its utility as a general balance and performance-based task. Single-leg vertical hopping demonstrated greater knee flexion and quadriceps activations, indicating its potential value for assessing quadriceps function and knee control under weight-bearing conditions during rehabilitation. Lastly, single-leg backward hopping was characterized by the lowest trunk and ankle flexion, the highest knee moments, and the lowest hip and ankle moments, along with elevated activation of the quadriceps and reduced activation of the hamstrings and triceps surae. These findings highlight backward hopping as a more knee-dominant task and support its potential clinical utilization as a sensitive and functionally specific assessment for identifying quadriceps deficits and evaluating knee function.

## Data Availability

The raw data supporting the conclusions of this article will be made available by the authors, without undue reservation.

## References

[1] Piussi R Simonson R Zsidai B Grassi A Karlsson J Della Villa F Better safe than sorry? A systematic review with meta-analysis on time to return to sport after Acl reconstruction as a risk factor for second Acl injury. J Orthop Sports Phys Ther. (2024) 54(3):161–75. 10.2519/jospt.2023.11977 38032099

[2] Tayfur B Charuphongsa C Morrissey D Miller SC. Neuromuscular function of the knee joint following knee injuries: does it ever get back to normal? A systematic review with meta-analyses. Sports Med. (2021) 51(2):321–38. 10.1007/s40279-020-01386-6 33247378 PMC7846527

[3] Rodriguez-Merchan EC Valentino LA. Return to sport activities and risk of reinjury following primary anterior cruciate ligament reconstruction. Arch Bone Jt Surg. (2022) 10(8):648–60. 10.22038/abjs.2021.50463.2504 36258743 PMC9569141

[4] Greenberg EM Greenberg ET Albaugh J Storey E Ganley TJ. Rehabilitation practice patterns following anterior cruciate ligament reconstruction: a survey of physical therapists. J Orthop Sports Phys Ther. (2018) 48(10):801–11. 10.2519/jospt.2018.8264 29787697

[5] Nagai T Schilaty ND Laskowski ER Hewett TE. Hop tests can result in higher limb symmetry Index values than isokinetic strength and leg press tests in patients following Acl reconstruction. Knee Surg Sports Traumatol Arthrosc. (2020) 28(3):816–22. 10.1007/s00167-019-05513-3 31025059 PMC6814513

[6] Ithurburn MP Longfellow MA Thomas S Paterno MV Schmitt LC. Knee function, strength, and resumption of preinjury sports participation in young athletes following anterior cruciate ligament reconstruction. J Orthop Sports Phys Ther. (2019) 49(3):145–53. 10.2519/jospt.2019.8624 30770031

[7] Krishnan C Johnson AK Palmieri-Smith RM. Mechanical factors contributing to altered knee extension moment during gait after Acl reconstruction: a longitudinal analysis. Med Sci Sports Exerc. (2022) 54(12):2208–15. 10.1249/mss.0000000000003014 35941516 PMC9669176

[8] Girdwood MA Crossley KM Rio EK Patterson BE Haberfield MJ Couch JL Hop to it! A systematic review and longitudinal meta-analysis of hop performance after Acl reconstruction. Sports Med. (2025) 55(1):101–13. 10.1007/s40279-024-02121-1 39414723 PMC11787245

[9] Losciale JM Zdeb RM Ledbetter L Reiman MP Sell TC. The association between passing return-to-sport criteria and second anterior cruciate ligament injury risk: a systematic review with meta-analysis. J Orthop Sports Phys Ther. (2019) 49(2):43–54. 10.2519/jospt.2019.8190 30501385

[10] Kotsifaki A Whiteley R Van Rossom S Korakakis V Bahr R Sideris V Single leg hop for distance symmetry masks lower limb biomechanics: time to discuss hop distance as decision criterion for return to sport after acl reconstruction? Br J Sports Med. (2022) 56(5):249–56. 10.1136/bjsports-2020-103677 33687928

[11] Kotsifaki A Van Rossom S Whiteley R Korakakis V Bahr R Sideris V Single leg vertical jump performance identifies knee function deficits at return to sport after Acl reconstruction in male athletes. Br J Sports Med. (2022) 56(9):490–8. 10.1136/bjsports-2021-104692 35135826 PMC9016240

[12] Fischer F Blank C Dünnwald T Gföller P Herbst E Hoser C Isokinetic extension strength is associated with single-leg vertical jump height. Orthop J Sports Med. (2017) 5(11):2325967117736766. 10.1177/2325967117736766 29147670 PMC5672995

[13] Laudner K Evans D Wong R Allen A Kirsch T Long B Relationship between isokinetic knee strength and jump characteristics following anterior cruciate ligament reconstruction. Int J Sports Phys Ther. (2015) 10(3):272–80. 26075142 PMC4458914

[14] Song Y Salsgiver L Van Valkenburg K Christofferson N Lo Y Feng Z Hopping backward to move forward: single-leg backward hopping can better detect decreased quadriceps strength induced by a fatigue protocol compared to forward and vertical hopping. J Sport Health Sci. (2024) 14:100976. 10.1016/j.jshs.2024.100976 39237062 PMC11863272

[15] Song Y Nguyen T Gu Y Su W Malik N. The effect of arm swings on lower limb kinetics during single-leg forward, vertical, and backward hopping. J Biomech. (2025) 183:112605. 10.1016/j.jbiomech.2025.112605 40058019

[16] Adams D Logerstedt DS Hunter-Giordano A Axe MJ Snyder-Mackler L. Current concepts for anterior cruciate ligament reconstruction: a criterion-based rehabilitation progression. J Orthop Sports Phys Ther. (2012) 42(7):601–14. 10.2519/jospt.2012.3871 22402434 PMC3576892

[17] Sasaki K Neptune RR. Individual muscle contributions to the Axial knee joint contact force during normal walking. J Biomech. (2010) 43(14):2780–4. 10.1016/j.jbiomech.2010.06.011 20655046 PMC2963724

[18] Boden BP Sheehan FT. Mechanism of non-contact Acl injury: oref clinical research award 2021. J Orthop Res. (2022) 40(3):531–40. 10.1002/jor.25257 34951064 PMC8858885

[19] Englander ZA Foody JN Cutcliffe HC Wittstein JR Spritzer CE DeFrate LE. Use of a novel multimodal imaging technique to model *in vivo* quadriceps force and Acl strain during dynamic activity. Am J Sports Med. (2022) 50(10):2688–97. 10.1177/03635465221107085 35853157 PMC9875882

[20] van Melick N van der Weegen W van der Horst N. Quadriceps and hamstrings strength reference values for athletes with and without anterior cruciate ligament reconstruction who play popular pivoting sports, including soccer, basketball, and handball: a scoping review. J Orthop Sports Phys Ther. (2022) 52(3):142–55. 10.2519/jospt.2022.10693 34972481

[21] Turk R Shah S Chilton M Thomas TL Anene C Mousad A Return to sport after anterior cruciate ligament reconstruction requires evaluation of >2 functional tests, psychological readiness, quadriceps/hamstring strength, and time after surgery of 8 months. Arthroscopy. (2023) 39(3):790–801.e6. 10.1016/j.arthro.2022.08.038 36216133

[22] Fukashiro S Besier TF Barrett R Cochrane J Nagano A Lloyd DG. Direction control in standing horizontal and vertical jumps. Int J Sport Health Sci. (2005) 3(Special_Issue_2005):272–9. 10.5432/ijshs.3.272

[23] Clemente de Oliveira HL Moreira PVS Menegaldo LL. Biomechanical differences between horizontal and vertical single-leg jumps: what could each one reveal about functional impairments? Sports Biomech. (2025):1–17. 10.1080/14763141.2025.2557396 40981494

[24] Hermens HJ Freriks B Merletti R Stegeman DF Blok JH Rau G (editors) European Recommendations for Surface Electromyography: Results of the Seniam Project. Enschede: Roessingh Research and Development (1999).

[25] Schwartz C Wang FC Forthomme B Denoël V Brüls O Croisier JL. Normalizing gastrocnemius muscle Emg signal: an optimal set of Maximum voluntary isometric contraction tests for young adults considering reproducibility. Gait Posture. (2020) 82:196–202. 10.1016/j.gaitpost.2020.08.129 32937272

[26] Song Y Goršič M Feng Z Cordova H Li L Dai B Effects of a back-assist exosuit in lab-based approximations of construction tasks performed by novices and experienced construction workers. Ergonomics. (2025) 68(2):267–84. 10.1080/00140139.2024.2325535 39387502

[27] Kristianslund E Krosshaug T van den Bogert AJ. Effect of low pass filtering on joint moments from inverse dynamics: implications for injury prevention. J Biomech. (2012) 45(4):666–71. 10.1016/j.jbiomech.2011.12.011 22227316

[28] Song Y Li L Layer J Fairbanks R Hughes G Smith D Unanticipated mid-flight external trunk perturbation increased frontal plane acl loading variables during sidestep cuttings. J Sports Sci. (2024) 42(7):599–610. 10.1080/02640414.2024.2353404 38734986 PMC11157851

[29] Cole GK Nigg BM Ronsky JL Yeadon MR. Application of the joint coordinate system to three-dimensional joint attitude and movement representation: a standardization proposal. J Biomech Eng. (1993) 115(4a):344–9. 10.1115/1.2895496 8309227

[30] Bresler B Frankel JP. The forces and moments in the leg during level walking. TransAm Soc Mech Eng. (2022) 72(1):27–36. 10.1115/1.4016578

[31] Kotsifaki A Korakakis V Graham-Smith P Sideris V Whiteley R. Vertical and horizontal hop performance: contributions of the hip, knee, and ankle. Sports Health. (2021) 13(2):128–35. 10.1177/1941738120976363 33560920 PMC8167345

[32] Benjamini Y Hochberg Y. Controlling the false discovery rate: a practical and powerful approach to multiple testing. J R Stat Soc Ser (Methodological). (1995) 57(1):289–300. 10.1111/j.2517-6161.1995.tb02031.x

[33] Cohen J. Statistical Power Analysis for the Behavioral Sciences. 2nd ed. Hillsdale: Routledge (1988).

[34] Ciccodicola EM Hanson AM Roberts SE Katzel MJ Wren TAL. Biomechanics and performance of single-leg vertical and horizontal hop in adolescents post-anterior cruciate ligament reconstruction. Biomechanics. (2025) 5(1):5. 10.3390/biomechanics5010005

[35] Donohue MR Ellis SM Heinbaugh EM Stephenson ML Zhu Q Dai B. Differences and correlations in knee and hip mechanics during single-leg landing, single-leg squat, double-leg landing, and double-leg squat tasks. Res Sports Med. (2015) 23(4):394–411. 10.1080/15438627.2015.1076413 26275102

[36] Zarro MJ Stitzlein MG Lee JS Rowland RW Gray VL Taylor JB Single-Leg vertical hop test detects greater limb asymmetries than horizontal hop tests after anterior cruciate ligament reconstruction in ncaa division 1 collegiate athletes. Int J Sports Phys Ther. (2021) 16(6):1405–14. 10.26603/001c.29595 34909247 PMC8637251

[37] Song Y Li L Jensen MA Dai B. Jump-Landing kinetic asymmetries persisted despite symmetric squat kinetics in collegiate athletes following anterior cruciate ligament reconstruction. Sports Biomech. (2025) 24(4):999–1012. 10.1080/14763141.2023.2207552 37144626 PMC10625647

[38] de Leva P. Adjustments to zatsiorsky-seluyanov's segment inertia parameters. J Biomech. (1996) 29(9):1223–30. 10.1016/0021-9290(95)00178-6 8872282

[39] Hara M Shibayama A Takeshita D Hay DC Fukashiro S. A comparison of the mechanical effect of arm swing and countermovement on the lower extremities in vertical jumping. Hum Mov Sci. (2008) 27(4):636–48. 10.1016/j.humov.2008.04.001 18674837

