# Supplementary material for: Lower limb muscle activation and biomechanics during single-leg hopping in different directions
Source: Front Sports Act Living. 2026 Jan 9;7:1733669. doi: 10.3389/fspor.2025.1733669 (PMC12827753; doi:10.3389/fspor.2025.1733669)
Supplement: Supplementary file 1 [file Table1.docx]

Appendix S1. Mean ± standard deviation and main effect p-values of repeated-measures ANOVAs in muscle activation variables.

|  | Single-leg forward hopping | Single-leg vertical hopping | Single-leg backward hopping | P-values of main effect |
| --- | --- | --- | --- | --- |
| Mean muscle activation during jumping | | | | |
| Vastus lateralis (%) | 1.38 ± 1.03 ^b^ | 2.04 ± 1.58 ^a^ | 1.94 ± 1.50 ^a^ | **<0.001** |
| Rectus femoris (%) | 0.29 ± 0.18 ^b^ | 0.53 ± 0.21 ^a^ | 0.52 ± 0.22 ^a^ | **<0.001** |
| Vastus medialis (%) | 1.57 ± 1.11 ^c^ | 2.17 ± 1.85 ^a^ | 2.04 ± 1.81 ^b^ | **<0.001** |
| Biceps femoris (%) | 0.23 ± 0.10 ^a^ | 0.13 ± 0.08 ^b^ | 0.11 ± 0.07 ^c^ | **<0.001** |
| Semitendinosus (%) | 0.20 ± 0.08 ^a^ | 0.10 ± 0.06 ^b^ | 0.09 ± 0.06 ^b^ | **<0.001** |
| Gastrocnemius lateralis (%) | 0.65 ± 0.25 ^a^ | 0.60 ± 0.21 ^b^ | 0.56 ± 0.22 ^b^ | **<0.001** |
| Gastrocnemius medialis (%) | 0.73 ± 0.32 ^a^ | 0.66 ± 0.28 ^b^ | 0.56 ± 0.24 ^c^ | **<0.001** |
| Soleus (%) | 0.84 ± 0.41 ^a^ | 0.84 ± 0.39 ^a^ | 0.71 ± 0.35 ^b^ | **<0.001** |
| Peak muscle activation during jumping | | | | |
| Vastus lateralis (%) | 3.58 ± 2.69 ^b^ | 4.09 ± 3.14 ^a^ | 3.99 ± 3.14 ^a^ | **0.001** |
| Rectus femoris (%) | 0.74 ± 0.42 ^b^ | 1.12 ± 0.51 ^a^ | 1.07 ± 0.47 ^a^ | **<0.001** |
| Vastus medialis (%) | 4.14 ± 3.13 | 4.28 ± 3.30 | 4.30 ± 3.90 | 0.536 |
| Biceps femoris (%) | 0.53 ± 0.22 ^a^ | 0.35 ± 0.22 ^b^ | 0.29 ± 0.18 ^c^ | **<0.001** |
| Semitendinosus (%) | 0.50 ± 0.30 ^a^ | 0.27 ± 0.17 ^b^ | 0.31 ± 0.22 ^b^ | **<0.001** |
| Gastrocnemius lateralis (%) | 1.26 ± 0.57 | 1.21 ± 0.44 | 1.15 ± 0.58 | 0.247 |
| Gastrocnemius medialis (%) | 1.23 ± 0.53 ^a^ | 1.19 ± 0.49 ^a^ | 1.02 ± 0.47 ^b^ | **<0.001** |
| Soleus (%) | 1.76 ± 0.87 ^a^ | 1.88 ± 0.93 ^a^ | 1.44 ± 0.75 ^b^ | **<0.001** |

Note. a, b, and c, significantly different among the three hopping directions: a is the greatest, b is the second greatest, and c is the least.

Appendix S2. Effect sizes (p-values) for paired comparisons of muscle activations showing significant main effects among single-leg hopping tasks.

|  | Single-leg forward vs. vertical hopping | Single-leg forward vs. backward hopping | Single-leg vertical vs. backward hopping |
| --- | --- | --- | --- |
| Mean muscle activation during jumping | | | |
| Vastus lateralis (%) | **0.83 (<0.001)** | **0.82 (<0.001)** | 0.27 (0.070) |
| Rectus femoris (%) | **1.83 (<0.001)** | **1.34 (<0.001)** | 0.14 (0.347) |
| Vastus medialis (%) | **0.73 (<0.001)** | **0.58 (<0.001)** | **0.39 (0.010)** |
| Biceps femoris (%) | **1.51 (<0.001)** | **1.32 (<0.001)** | **0.55 (<0.001)** |
| Semitendinosus (%) | **1.63 (<0.001)** | **1.38 (<0.001)** | 0.13 (0.373) |
| Gastrocnemius lateralis (%) | **0.38 (0.012)** | **0.53 (0.001)** | 0.28 (0.062) |
| Gastrocnemius medialis (%) | **0.45 (0.003)** | **0.91 (<0.001)** | **0.92 (<0.001)** |
| Soleus (%) | 0.02 (0.904) | **0.66 (<0.001)** | **0.62 (<0.001)** |
| Peak muscle activation during jumping | | | |
| Vastus lateralis (%) | **0.49 (0.001)** | **0.41 (0.007)** | 0.12 (0.393) |
| Rectus femoris (%) | **1.46 (<0.001)** | **0.95 (<0.001)** | 0.23 (0.113) |
| Biceps femoris (%) | **1.15 (<0.001)** | **1.19 (<0.001)** | **0.35 (0.019)** |
| Semitendinosus (%) | **1.05 (<0.001)** | **0.64 (<0.001)** | 0.19 (0.192) |
| Gastrocnemius medialis (%) | 0.19 (0.189) | **0.68 (<0.001)** | **0.89 (<0.001)** |
| Soleus (%) | 0.24 (0.102) | **0.71 (<0.001)** | **0.67 (<0.001)** |

Appendix S3. Effect sizes (p-values) of paired comparisons among single-leg hopping tasks in biomechanical parameters.

|  | Single-leg forward vs. vertical hopping | Single-leg forward vs. backward hopping | Single-leg vertical vs. backward hopping |
| --- | --- | --- | --- |
| Hopping performance (m) | **4.14 (<0.001)** | **2.58 (<0.001)** | **4.40 (<0.001)** |
| Jumping duration (s) | **0.71 (<0.001)** | **0.38 (0.011)** | **1.15 (<0.001)** |
| Peak trunk flexion angle during jumping (°) | **0.92 (<0.001)** | **1.69 (<0.001)** | **0.79 (<0.001)** |
| Peak hip flexion angle during jumping (°) | **0.67 (<0.001)** | **0.79 (<0.001)** | 0.01 (0.971) |
| Peak knee flexion angle during jumping (°) | **1.51 (<0.001)** | **0.86 (<0.001)** | **0.72 (<0.001)** |
| Peak ankle dorsiflexion angle during jumping (°) | **1.21 (<0.001)** | **2.88 (<0.001)** | **3.35 (<0.001)** |
| Peak hip moment during jumping (BW*BH) | **0.78 (<0.001)** | **1.20 (<0.001)** | **0.60 (<0.001)** |
| Peak knee moment during jumping (BW*BH) | **1.78 (<0.001)** | **2.14 (<0.001)** | **1.07 (<0.001)** |
| Peak ankle moment during jumping (BW*BH) | **2.82 (<0.001)** | **3.23 (<0.001)** | **1.44 (<0.001)** |

Note. BW: body weight; BH: body height.
